# Characteristic retinal atrophy pattern allows differentiation between pediatric MOGAD and MS after a single optic neuritis episode

**DOI:** 10.1007/s00415-022-11256-y

**Published:** 2022-07-23

**Authors:** T. Pakeerathan, J. Havla, C. Schwake, A. Salmen, S. Bigi, M. Abegg, D. Brügger, T. Ferrazzini, A.-K. Runge, M. Breu, B. Kornek, G. Bsteh, A. Felipe-Rucián, M. Ringelstein, O. Aktas, M. Karenfort, E. Wendel, I. Kleiter, K. Hellwig, T. Kümpfel, C. Thiels, T. Lücke, R. Gold, K. Rostasy, I. Ayzenberg

**Affiliations:** 1 Department of Neurology, St. Josef-Hospital, Ruhr-University Bochum, 44791 Bochum, Germany; 2 Institute of Clinical Neuroimmunology, LMU Hospital, Ludwig-Maximilians Universität München, Munich, Germany; 3 Data Integration for Future Medicine (DIFUTURE) Consortium, LMU Hospital, Ludwig-Maximilians Universität München, Munich, Germany; 4 Department of Neurology, Inselspital, Bern University Hospital, University of Bern, Bern, Switzerland; 5 Institute for Social and Preventive Medicine, University of Bern, Bern, Switzerland; 6 Division of Child Neurology, Department of Pediatrics, University Children’s Hospital Bern, University of Bern, Bern, Switzerland; 7 Department of Ophthalmology, Inselspital, Bern University Hospital, University of Bern, Bern, Switzerland; 8 Division of Pediatric Pulmonology, Allergology and Endocrinology, Department of Pediatrics and Adolescent Medicine, Medical University of Vienna, Vienna, Austria; 9 Department of Neurology, Medical University of Vienna, Vienna, Austria; 10 Department of Pediatric Neurology, Universitat Autònoma de Barcelona, Vall d’Hebron Hospital, Barcelona, Spain; 11 Department of Neurology, Medical Faculty, Heinrich-Heine-University Düsseldorf, Düsseldorf, Germany; 12 Department of Neurology, Center for Neurology and Neuropsychiatry, LVR-Klinikum, Heinrich-Heine-University Düsseldorf, Düsseldorf, Germany; 13 Department of General Paediatrics, Neonatology and Paediatric Cardiology, Heinrich-Heine-University Düsseldorf, Düsseldorf, Germany; 14 Department of Pediatric Neurology, Olgahospital, Stuttgart, Germany; 15 Marianne-Strauß-Klinik, Behandlungszentrum Kempfenhausen für Multiple Sklerose Kranke, Berg, Germany; 16 Department of Neuropediatrics, University Children’s Hospital, Ruhr-University, Bochum, Germany; 17 Department of Pediatric Neurology, Children’s Hospital Datteln, University Witten/Herdecke, Witten, Germany

**Keywords:** Optical coherence tomography, Visual evoked potential, Optic neuritis, Myelin-oligodendrocyte-glycoprotein IgG, MOGAD, Multiple sclerosis, Pediatric patients, Children

## Abstract

**Background:**

Optic neuritis (ON) is the most prevalent manifestation of pediatric multiple sclerosis (MS^ped^) and myelin-oligodendrocyte glycoprotein antibody-associated disease (MOGAD^ped^) in children > 6 years. In this study, we investigated retinal atrophy patterns and diagnostic accuracy of optical coherence tomography (OCT) in differentiating between both diseases after the first ON episode.

**Methods:**

Patients were retrospectively identified in eight tertial referral centers. OCT, VEP and high/low-contrast visual acuity (HCVA/LCVA) have been investigated > 6 months after the first ON. Prevalence of pathological OCT findings was identified based on data of 144 age-matched healthy controls.

**Results:**

Thirteen MOGAD^ped^ (10.7 ± 4.2 years, F:M 8:5, 21 ON eyes) and 21 MS^ped^ (14.3 ± 2.4 years, F:M 19:2, 24 ON eyes) patients were recruited. We observed a significantly more profound atrophy of both peripapillary and macular retinal nerve fiber layer in MOGAD^ped^ compared to MS^ped^ (pRNFL global: 68.2 ± 16.9 vs. 89.4 ± 12.3 µm, *p* < 0.001; mRNFL: 0.12 ± 0.01 vs. 0.14 ± 0.01 mm^3^, *p* < 0.001). Neither other macular layers nor P100 latency differed. MOGAD^ped^ developed global atrophy affecting all peripapillary segments, while MS^ped^ displayed predominantly temporal thinning. Nasal pRNFL allowed differentiation between both diseases with the highest diagnostic accuracy (AUC = 0.902, cutoff < 62.5 µm, 90.5% sensitivity and 70.8% specificity for MOGAD^ped^). OCT was also substantially more sensitive compared to VEP in identification of ON eyes in MOGAD (pathological findings in 90% vs. 14%, *p* = 0.016).

**Conclusion:**

First MOGAD-ON results in a more severe global peripapillary atrophy compared to predominantly temporal thinning in MS-ON. Nasal pRNFL allows differentiation between both diseases with the highest accuracy, supporting the additional diagnostic value of OCT in children with ON.

## Introduction

Multiple sclerosis (MS) and myelin-oligodendrocyte glycoprotein (MOG) antibody-associated disease (MOGAD) are two major autoimmune-inflammatory demyelinating diseases of the central nervous system (CNS) associated with optic neuritis (ON) in children. ON represents the onset symptom of pediatric MS in 20–25% of cases, while 42% of all children with isolated ON later develop MS [1–3]. At the same time, ON is the most prevalent clinical manifestation of MOGAD in children > 6 years of age, while up to 45% of all children with isolated ON and 73% of those with bilateral ON are seropositive for MOG immunoglobulin(Ig)G [4–6]. The overlapping clinical manifestation makes an early and precise recognition of the underlying disease difficult. However, a correct diagnosis is therapeutically highly relevant. Pathophysiological mechanisms and recommended immunotherapies differ between both entities and classical MS medications can be ineffective or even worsen the course of MOGAD [7]. Testing for specific conformation-dependent autoantibodies, targeting MOG, requires transfected cell-based assays (CBA) and can be challenging. Low titers of MOG-IgG in serum have low positive predictive value and can be found in a number of other neurological conditions and even in healthy persons [8, 9]. At the same time a Japanese group recently reported a cohort with an intrathecal origin of MOG-IgG, in which 29% of patients were seropositive in CSF only [10, 11]. Considering these limitations and the unavailability of MOG-IgG CBA in many countries, an additional paraclinical biomarker for MOGAD could be diagnostically valuable.

ON results in a thinning of the retinal nerve fiber layer (RNFL) and the ganglion cell layer (GCL), that can be precisely evaluated by spectral-domain optical coherence tomography (OCT). While a moderate RNFL thinning, occurring predominantly in the temporal peripapillary segment, is typical for MS-ON, no specific atrophy pattern has been identified in MOGAD-ON, so far [12]. Available studies mostly reported profound retinal changes, probably due to a highly recurrent course of MOGAD-ON [13, 14]. Given the relative rarity and novelty of MOGAD, there are no studies directly comparing structural retinal changes and functional integrity of the visual pathway in pediatric MOGAD-ON and MS-ON.

Main objectives of this study were as follows: (1) to investigate pattern of the retinal neuroaxonal damage and persisting visual evoked potential (VEP) changes after a single ON episode in pediatric MOGAD, and (2) to evaluate the sensitivity and specificity of OCT and VEP parameters in differentiating between MOGAD-ON and MS-ON in children.

## Subjects and methods

### Study population

**Fig. 1:**
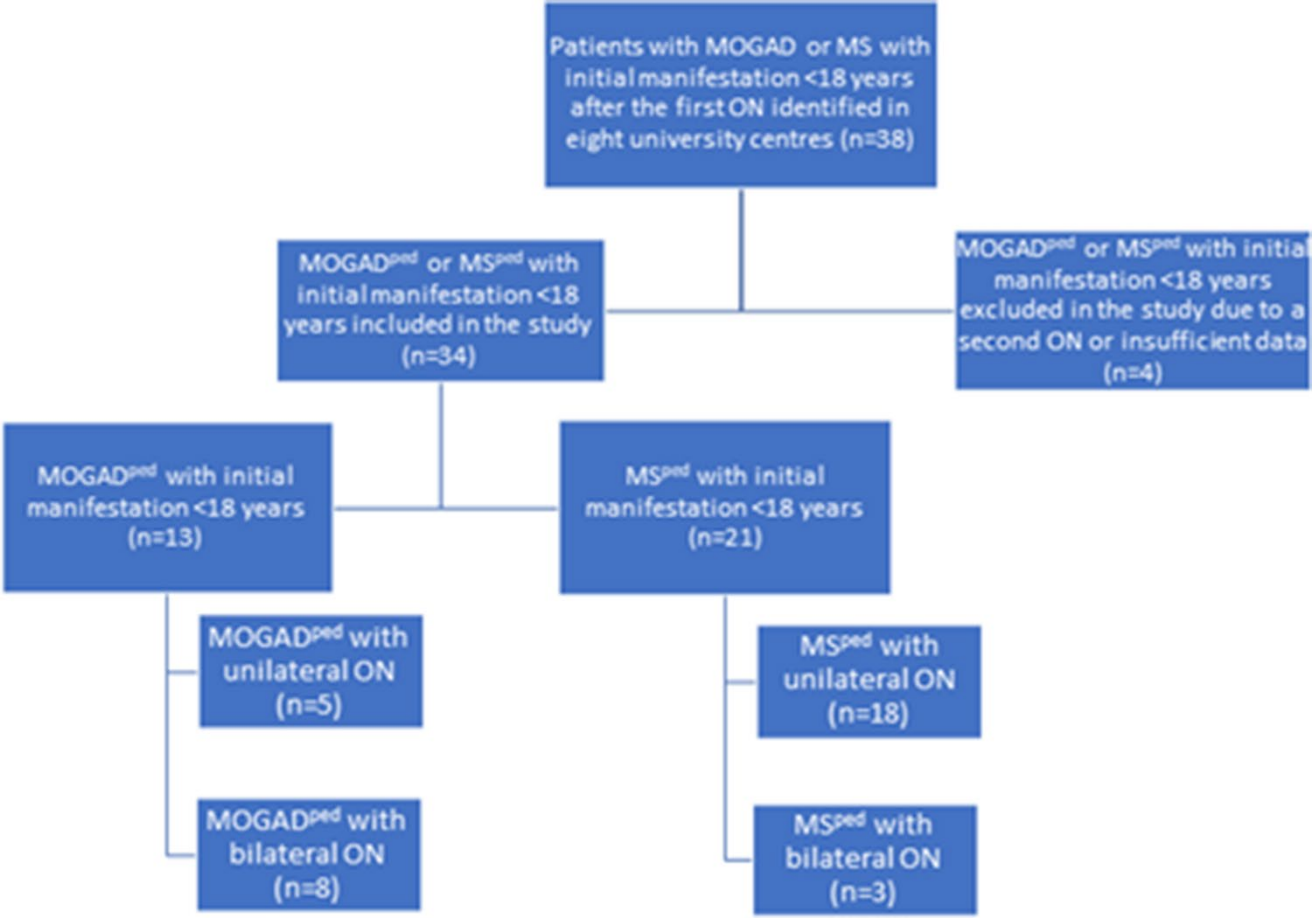
Flow chart of patients included in the study. During the study period, 38 pediatric MOGAD or MS patients after the first ON attack were identified in the participating centers. Four patients were excluded due to second ON attack during the first 6 months after the initial event. Depending on the diagnosis, the pediatric patients were divided into 2 groups: group (1) 13 MOG-IgG-patients with initial manifestation < 18 years (MOGAD^paed^) and group (2) 21 MS patients with initial manifestation < 18 years (MS^ped^)

In our multi-center, retrospective cross-sectional study, following Strengthening the Reporting of Observational Studies in Epidemiology (STROBE) statement guidelines [15], we analyzed the clinical and imaging data of pediatric MOGAD (MOGAD^ped^) and pediatric MS patients (MS^ped^) after the first ON episode, unilateral or bilateral ON, fulfilling the following inclusion criteria: (1) MOG-IgG positive status (> 1:32 in fixed or > 1:160 in live CBA) or diagnosis of MS based on McDonald criteria 2017 [16]; (2) age at first ON < 18 years of age;. 3. OCT and VEP performed > 6 months after the first ON episode. Patients with concomitant ophthalmological diseases, with positive aquaporin-4 IgG or with subsequent ON episodes before OCT were excluded from the analysis. Patients were recruited from 2018 to 2021 at eight specialized neuroimmunological university and non-university tertiary care centers (Department of Neurology, St. Josef-Hospital Bochum and University Children’s Hospital Bochum, Germany, *N* = 17, Children’s Hospital Datteln, *N* = 7, Institute of Clinical Neuroimmunology, LMU Hospital, Munich, Germany, *N* = 2, Department of Neurology, Medical Faculty, Heinrich-Heine-University, Düsseldorf, Germany, *N* = 3, Pediatric Neurology, Medical University of Vienna, Austria, *N* = 3, Departments of Neurology and Neuropediatrics, Inselspital, Bern University Hospital, Bern, Switzerland, *N* = 4 and Institute of Paediatrics, University Hospital Vall d’Hebron, Barcelona, Spain, *N* = 2, Fig. 1 with flow chart visualizing the recruitment process). Serum samples from all patients were analyzed for MOG-IgG and aquaporin-4-IgG during initial workup at least once by established cell-based assays at the discretion of each center using the laboratory’s cutoffs (MOG IFT, EUROIMMUN, Laboratory Prof. Stöcker, Germany; Laboratory Prof. Reindl, Medical University of Innsbruck, Innsbruck, Austria; Laboratory Prof. Meinl, LMU Hospital, Munich) [7, 17]. Demographical (sex and age at initial manifestation) and main clinical data (disease duration, number and side of clinical ON episode) were acquired for all patients. In addition, habitually corrected high-contrast and low-contrast monocular visual acuity (VA) was acquired using high-contrast and 2.5% low-contrast Sloan letter charts placed in a retro-illuminated light box at 2 m distance. Each chart consists of 14 lines with 5 letters per line that are standardized with equal difficulty per line and equal spacing between the lines. The total number of correct letters identified on each chart was tested to determine high and low-contrast VA (HCVA, LCVA; maximum: 70 letters). LCVA is data available for 11 MOGAD-ON eyes and 14 MS-ON eyes. Ethics approval was obtained from the Institutional Review Board of the Ruhr-University Bochum (#18-6397). The study was performed according to the Declaration of Helsinki (1964) in its currently applicable version.

### Optical coherence tomography (OCT)

The spectral-domain optical coherence tomography (SD-OCT, SPECTRALIS, Heidelberg Engineering, Heidelberg, Germany) with automatic real-time (ART) averaging was used in all participating centers. Based on local protocols a scan around the optic nerve head with an activated eye tracker (12°, 3.5 mm ring, 50 ≤ ART ≤ 100) and a macular volume scan (20° × 20°, 25 vertical B-scans, 20 ≤ ART ≤ 49) as a cylinder of 3 mm diameter around the fovea were performed. In the analysis, the thickness of the peripapillary retinal nerve fiber layer (pRNFL) and the volumes of the macular retinal nerve fiber layer (mRNFL), the combined ganglion cell and inner plexiform layer (GCIPL), the inner nuclear layer (INL), the combined outer plexiform and outer nuclear layer (OPONL), as well as the total macular volume (TMV) were included. The segmentation of all layers was performed semi-automatically using software from the SD-OCT manufacturer (Eye Explorer 1.9.10.0 with viewing module 6.3.4.0, Heidelberg Engineering, Heidelberg, Germany). All scans were carefully checked by experienced evaluators for sufficient quality and segmentation errors and were corrected if necessary. The SD-OCT data were analyzed and reported according to the recommendations of APOSTEL and OSCAR-IB [18, 19].

### Visual evoked potentials (VEP)

VEP data (Keypoint.net, Neurolite Software, Natus, Switzerland) were acquired according to International Society for Clinical Electrophysiology of Vision standards [20]. The data were collected from occipital midline referred to the mid-frontal electrode. Pattern reversal VEP was produced by high-contrast, black and white checks. The examination was performed in a dark room in 2 m distance. P100 latency and the P100-N125 amplitude were collected (latency in milliseconds, amplitude in mV). VEP data are available for patients who were seen in Bochum (data available for 11 MOGAD-ON eyes and 16 MS-ON eyes).

### Statistical methods

Clinical data, OCT and VEP results were compared between both groups (MOGAD^ped^ vs MS^ped^). Mean and standard deviation (SD) were calculated for continuous variables, frequency, and proportion for categorial variables. The non-parametric Mann–Whitney-*U*-Test and Chi-square-test were used to compare two independent groups. Statistical significance was defined as *p* < 0.05.

To describe pattern of retinal changes after ON precisely, we also reported frequencies of significant atrophy in different pRNFL quadrants and macular sectors in every group, defined as a decrease below 2SD, compared to a group of age-matched healthy controls (*N* = 144, f:m 32:40, age 12.49 ± 2.18). Healthy controls have been investigated in St. Josef-Hospital Bochum previously and will be reported separately [21]. VEP latency delay was defined as 2SD below mean (105 ± 8 ms, cutoff: 121 ms). SD-OCT data, VEP data and HCVA/LCVA in eyes with ON were compared between the MOGAD^ped^ and MS^ped^ cohorts using generalized estimating equation models (GEE) to account for within-patient inter-eye correlation. The correlation matrix parameter was set to “exchangeable”. Significant parameters were further included into receiver operating characteristic analysis (ROC analysis) to determine their sensitivity and specificity in differentiating MOGAD^ped^ from MS^ped^. Parameters with an area under the curve (AUC) > 0.700 were suitable parameters. We used Youden index analysis to find optimal cutoff values. To compare the sensitivity of OCT and VEP regarding the identification of eyes with a history of MOGAD-ON, we used the McNemar Test. Data were analyzed with SPSS version 28 (IBM SPSS Statistics).

## Results

### Cohort description

Thirteen MOG-IgG-positive children (MOGAD^ped^, female:male 8:5, mean age at ON 10.7 ± 4.2 years, 21 ON eyes) and 21 MS children (MS^ped^, female: male 19:2, mean age at ON 14.3 ± 2.4 years, 24 ON eyes) with a single unilateral or bilateral ON episode in their history were included into analysis. The main demographic and clinical data of both groups are depicted in Table 1. Aquaporin-4 IgG was negative in all patients. All 21 MS children tested negative for MOG-IgG in serum and positive oligoclonal bands in CSF. MOGAD^ped^ patients were younger at ON onset compared to MS^ped^, whereas there was no significant difference in disease duration till OCT investigation in both groups. Simultaneous bilateral ON were significantly more prevalent in MOGAD^ped^ compared to MS^ped^ (*p* < 0.001). Seven of 13 MOG-IgG-positive children were initially diagnosed with ON only, 3 children with ADEM + ON, 2 children with NMOSD, and 1 child with encephalomyelitis + ON. Three patients in each group received plasma exchange, due to steroid refractory ON (5 MOGAD-ON eyes, 3 MS-ON eyes). A long-term immunotherapy was administered in 7 of 13 (53.8%) of MOGAD^ped^ (4 intravenous immunoglobulin/subcutaneous immunoglobulin (IVIG/SCIG), 1 monotherapy with oral prednisone, 1 rituximab, 1 dimethyl fumarate) and 18 of 21 (85.7%) MS^ped^ (6 ocrelizumab, 3 fingolimod, 2 rituximab, 2 interferon beta, 2 monotherapy with oral prednisone, 1 dimethyl fumarate, 1 natalizumab, 1 glatiramer acetate).

**Table 1:** Demographic and main clinical characteristics of pediatric MOGAD and MS cohorts

	MOGAD^ped^ (*n* = 13)	MS^ped^ (*n* = 21)	*p*-value
Age at initial disease manifestation, mean ± SD	9.0 ± 3.28	14.1 ± 2.5	< 0.001
Age at ON, mean ± SD	10.7 ± 4.2	14.3 ± 2.4	< 0.001
ON as first event, *n* (%)	8 (61.5%)	20 (95.2%)	< 0.001
Females, *n* (%)	8 (61.5%)	19 (90.5%)	0.010
Disease duration (in years), median (range)	3 (1–15)	3 (1–13)	0.382
Ethnicity, *n*	13/13 Caucasians	21/21 Caucasians	–
Patients with a simultaneous bilateral ON, *n* (%)	8 (61.5%)	3 (14.3%)	< 0.001
Total ON eyes, *n* (%)	21 (80.8%)	24 (57.1%)	0.045
Positive OCB, *n* (%)	0 (0%)	21 (100%)	–
HCVA, number of correctly stated letters, mean ± SD	54.3 ± 5.4	54.8 ± 7.7	0.483
2.5% LCVA, number of correctly stated letters, mean ± SD	24.3 ± 10.0	27.4 ± 11.0	0.472

*MOGAD* myelin oligodendrocyte glycoprotein-antibody-associated disease, *MS* multiple sclerosis, *MOGAD*^*ped*^ pediatric MOGAD patients, *MS*^*ped*^ pediatric MS patients, *ON* optic neuritis, *OCB* oligoclonal bands, *HCVA* high-contrast visual acuity, *LCVA* low-contrast visual acuity, *SD* standard deviation

### Different patterns of peripapillary retinal atrophy in MOGAD^ped^ and MS^ped^

**Fig. 2:**
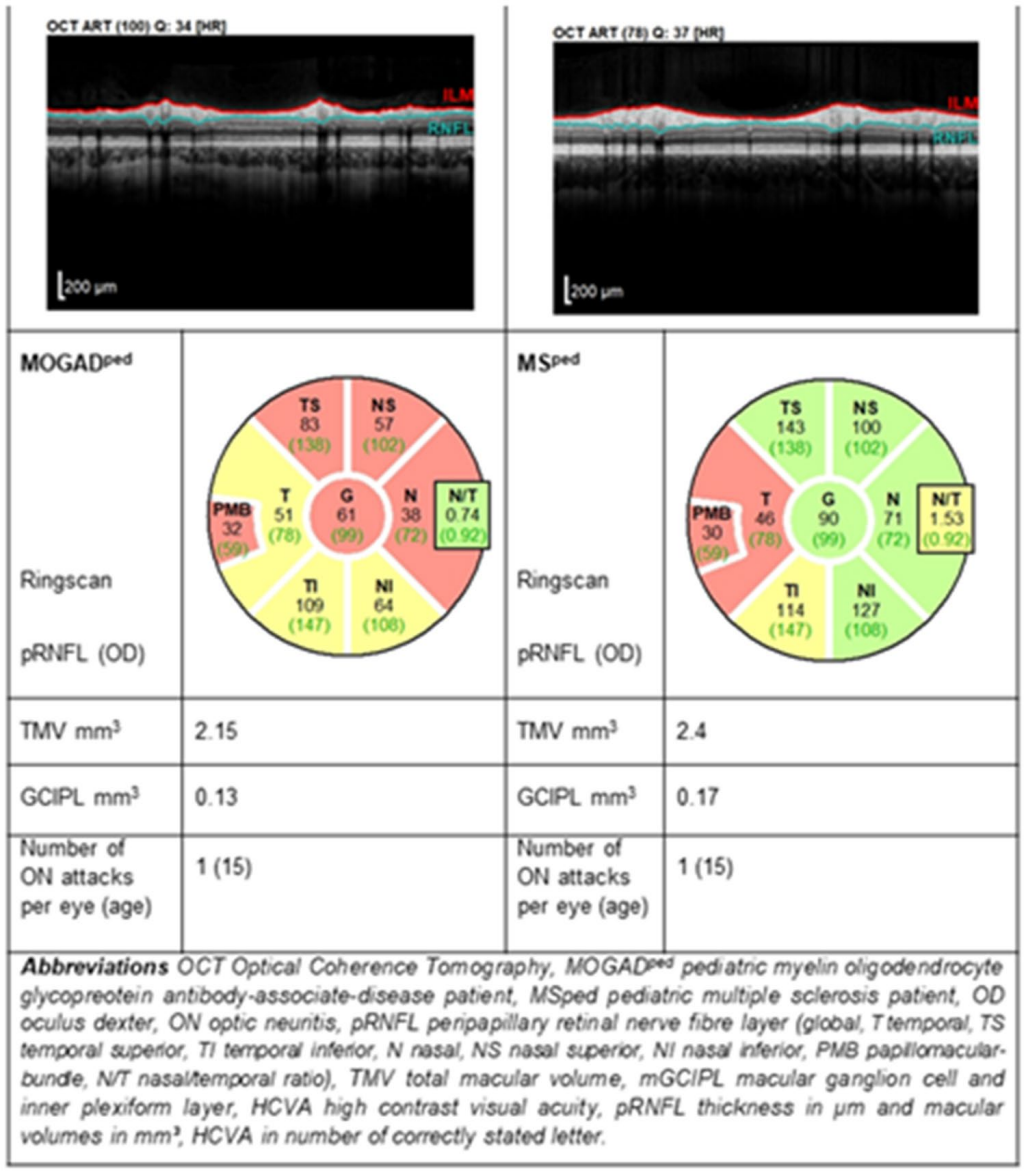
Substantial global pRNFL atrophy in MOGAD-ON compared to moderate predominantly temporal atrophy in MS-ON after a single ON episode

We observed a difference in the pattern of peripapillary retinal axonal degeneration between MOGAD^ped^ and MS^ped^ in ON affected eyes (Table 2). The frequency of marginal to severe atrophy comparing to healthy controls was in different pRNFL segments as following: temporal (T; 81.0 for MOGAD^ped^ vs 45.8% for MS^ped^), temporal superior (TS; 71.4 vs 38.1%), temporal inferior (TI; 81.0 vs 37.5%), nasal (N; 52.4 vs 0%), nasal superior (NS; 38.1 vs 8.3%) and nasal inferior (NI; 42.9 vs 0%) for MOGAD-ON and MS-ON eyes, accordingly. A direct comparison of both groups confirmed significantly more profound pRNFL atrophy in MOGAD^ped^ globally (pRNFL G) as well as in following segments (pRNFL TS, pRNFL TI, pRNFL N, pRNFL NS, pRNFL NI), whereas pRNFL T and the papillomacular bundle (PMB) were comparable (Fig. 2 with exemplary OCT images). Taken altogether, there was a mild-to-moderate temporal pRNFL thinning in MS-ON and a substantial global atrophy in MOGAD-ON already after the first ON episode.

**Table 2:** OCT and VEP measures after a single ON in MOGAD^ped^ and MS^ped^: direct comparison and prevalence of moderate to severe atrophy in each group compared to healthy controls

Parameter	MOGAD^ped^ with ON = 1 (21 eyes, mean ± SD)	MS^ped^ with ON = 1 (24 eyes, mean ± SD)	MOGAD^ped^ vs. MS^ped^ with ON = 1, *p*-value	Cutoff value (mean-2 SD, for VEP P100 latency: mean + 2SD)	MOGAD^ped^ with ON = 1 (21 eyes, number of pathological results)	MS^ped^ with ON = 1 (24 eyes, number of pathological results)
G pRNFL	68.2 ± 16.9	89.4 ± 12.3	** < 0.001**	85.7	19 (90.5%)*	10 (41.7%)
T pRNFL	47.7 ± 14.2	56.8 ± 17.0	0.096	54.3	17 (81.0%)	11 (45.8%)
TS pRNFL	98.0 ± 22.7	127.5 ± 36.0	**0.003**	114.5	15 (71.4%)	8 (38.1%)
TI pRNFL	99.7 ± 32.5	125.6 ± 23.2	**0.017**	117.9	17 (81.0%)	9 (37.5%)
N pRNFL	46.7 ± 11.1	66.9 ± 10.8	** < 0.001**	43.1	11 (52.4%)	0 (0.0%)
NS pRNFL	81.7 ± 26.5	109.4 ± 20.3	** < 0.001**	76.6	8 (38.1%)	2 (8.3%)
NI pRNFL	75.3 ± 24.9	102.7 ± 16.5	** < 0.001**	67.3	9 (42.9%)	0 (0.0%)
PMB	39.1 ± 14.3	42.4 ± 10.7	0.446	40.7	14 (66.7%)	11 (45.8%)
N/T Ratio	1.06 ± 0.34	1.28 ± 0.35	**0.039**	0.42 < x < 1.12	7 (33.3%)	14 (58.3%)*
TMV 3 mm	2.17 ± 0.15	2.25 ± 0.13	0.154	2.21	7 (33.3%)	9 (37.5%)
mRNFL 3 mm	0.12 ± 0.01	0.14 ± 0.01	** < 0.001**	0.13	13 (61.9%)	4 (16.7%)
GCIPL 3 mm	0.48 ± 0.09	0.54 ± 0.1	0.113	0.55	11 (52.4%)	13 (54.2%)
INL 3 mm	0.27 ± 0.03	0.27 ± 0.03	0.742	0.23	2 (10.0%)	1 (4.2%)
OPONL 3 mm	0.77 ± 0.06	0.74 ± 0.05	0.212	0.63	0 (0.0%)	0 (0.0%)
VEP P100 latency	118.7 ± 9.3	124.6 ± 15.2	0.249	121	3 (14.3%)	7(29.2%)

*Most sensitive parameters confirming history of the ON in each group

*MOGAD* myelin oligodendrocyte glycoprotein-antibody-associated disease, *MS* multiple sclerosis, *MOGAD*^*ped*^ pediatric MOGAD patients, *MS*^*ped*^ pediatric MS patients, *ON* optic neuritis, *MOGAD-ON* MOGAD patient’s eyes with a history of ON, *MS-ON* MS patient’s eyes with a history of ON, *pRNFL peripapillary retinal nerve* fiber layer (G global, T temporal, TS temporal superior, TI temporal inferior, N nasal, NS nasal superior, NI nasal inferior, PMB papillomacular bundle, N/T nasal/temporal ratio), *TMV* total macular volume, *mRNFL* macular retinal nerve fiber layer, *mGCIPL* macular ganglion cell and inner plexiform layer, *mINL* macular inner nuclear layer, *mOPONL* macular outer plexiform and outer inner nuclear layer

pRNFL thickness in µm and macular volumes in mm^3^, VEP P100 latency in ms

*p*-value: significant results *p* < 0.05 are indicated in bold letter

### Comparable changes in macular OCT, visual evoked potential, and visual acuity

There were neither significant difference in the total macular volume no in the GCIPL, the INL and the OPONL between both patient groups (Table 2). Similarly, the frequencies of significant pathological findings compared to healthy controls did not differ between MOGAD^ped^ and MS^ped^. The macular RNFL volume was the only parameter demonstrating a significant more profound thinning in MOGAD^ped^ vs MS^ped^.

VEP P100 latencies were also comparable in both groups, while a significant delay could be found in only 14.3% and 29.2% of MOGAD^ped^ and MS^ped^ ON eyes, respectively. There were no differences in the visual acuity between the groups (Table 2).

### Nasal pRNFL demonstrated highest accuracy in distinguishing MOGAD^ped^ and MS^ped^

All parameters demonstrating significant differences between both groups were included into the ROC analysis (Table 3). In the analysis of pRNFL segments nasal pRNFL demonstrated the highest diagnostic accuracy (area under the curve (AUC) = 0.902), followed by global pRNFL. Using a nasal pRNFL cutoff of 62.5 µm, we were able to distinguish between MOGAD^ped^ and MS^ped^ with a sensitivity of 90.5% and a specificity of 70.8%. Global pRNFL (AUC = 0.833, cutoff 67.9 µm) allowed a differentiation with a 61.9% sensitivity and 95.8% specificity accordingly. In the analysis of macular layers, only mRNFL (AUC = 0.839, cutoff 0.125 mm^3^) enabled a diagnostic distinction between both groups with a sensitivity of 81.3% and a specificity of 83.3%.

**Table 3:** Sensitivity and specificity of RNFL-parameters in distinguishing between pediatric MOGAD-ON and MS-ON

Parameter/segment	AUC	Cutoff (µm, mm^3^)	Sensitivity for MOGAD^ped^ vs MS^ped^ (%)	Specificity for MOGAD^ped^ vs MS^ped^ (%)
**pRNFL G**	**0.833**	**67.9**	**61.9**	**95.8**
pRNFL TS	0.799	118.5	81.0	66.7
pRNFL TI	0.775	110.5	81.0	70.8
**pRNFL N**	**0.902**	**62.5**	**90.5**	**70.8**
pRNFL NS	0.791	88.0	61.9	87.5
pRNFL NI	0.801	85.5	66.7	87.5
**mRNFL**	**0.839**	**0.125**	**81.3**	**83.3**

*MOGAD* myelin oligodendrocyte glycoprotein-antibody-associated disease, *MS* multiple sclerosis, *MOGAD*^*ped*^ pediatric MOGAD patients, *MS*^*ped*^ pediatric MS patients, *ON* optic neuritis, *MOGAD-ON* MOGAD patient’s eyes with a history of ON, *MS-ON* MS patient’s eyes with a history of ON, *pRNFL* peripapillary retinal nerve fiber layer (G global, TS temporal superior, TI temporal inferior, N nasal, NS nasal superior, NI nasal inferior), *mRNFL* macular RNFL, *AUC* area under the curve

pRNFL thickness in µm and macular volume in mm^3^

Only parameters with AUC > 0.700 were suitable parameters which are listed in the table. Parameter with the highest sensitivity and specificity indicated in bold letter

**Fig. 3:**
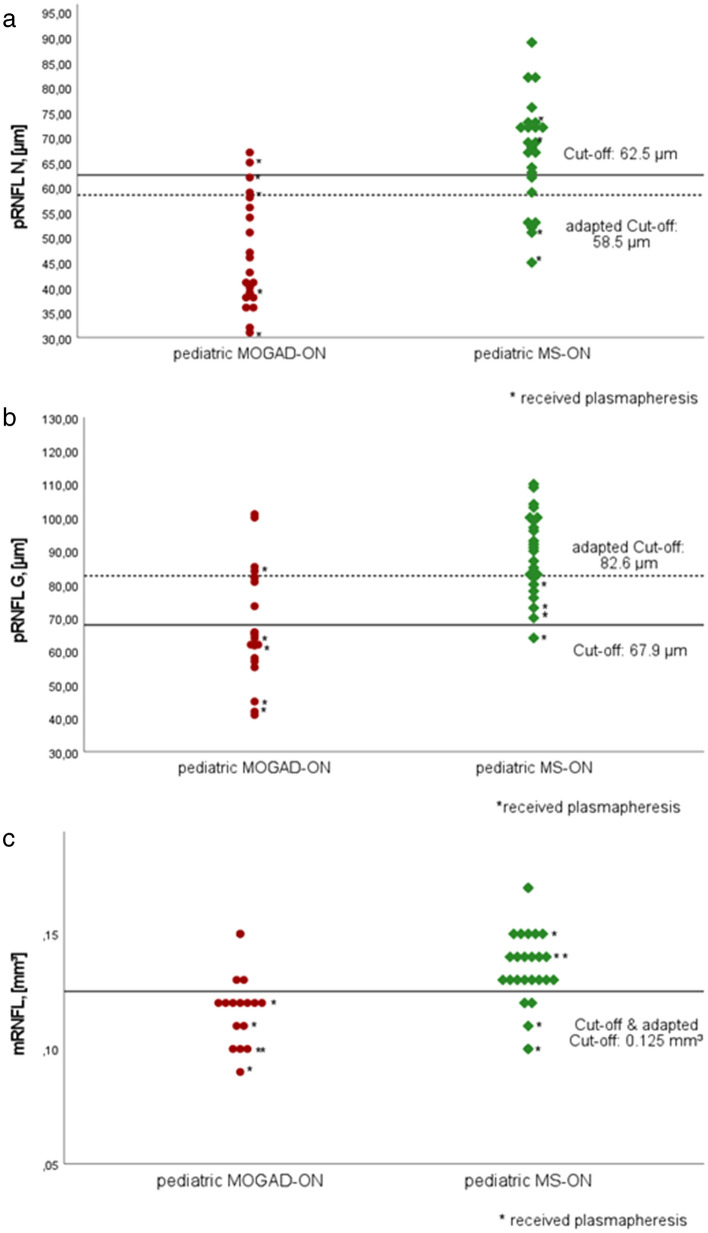
Scatterplot showing the distribution of **a** pRNFL N thickness, **b** pRNFL G thickness and **c** mRNFL volume. Optimal cutoff values for each feature were defined via the Youden Index in the ROC curves. Cutoffs are demonstrated for the whole group and adapted (dashed line) after excluding patients with plasma exchange from both groups

Concerning potential selection bias (recruitment of patients with most severe refractory ON in specialized neuroimmunological in-patient departments) and probable role of so far unknown autoantibodies, we performed an additional ROC analysis excluding patients treated with plasma exchange from both groups. In this subgroup, including 16 MOGAD-ON and 20 MS-ON eyes, the same 3 parameters had the best diagnostic accuracy, demonstrating even higher sensitivity and specificity in distinguishing between both diseases (nasal pRNFL AUC = 0.946, cutoff 58.5 µm, sensitivity of 93.8%, specificity of 84.2%; global pRNFL AUC = 0.860, cutoff 82.6 µm, sensitivity of 81.3%, specificity of 84.2%; mRNFL AUC = 0.837, cutoff 0.125 mm^3^ sensitivity of 72.7%, specificity of 89.5% for MOGAD^ped^ vs. MS^ped^, Fig. 3 with scatter plots showing the distribution of these three parameters). The McNemar test showed that OCT is more sensitive in comparison to VEP in MOGAD-ON to identify eyes with history of ON (*p* = 0.016).

## Discussion

In this study, we investigated structural retinal and functional optic nerve changes after the first ON in pediatric MOGAD and MS and evaluated the eligibility of OCT and VEP to differentiate between these two clinical entities. Optic neuritis was the first disease manifestation in the majority of patients in both groups. As expected, children with MOGAD developed significantly more often simultaneous bilateral ON, while 86% of those with MS had an unilateral involvement [5, 22]. The functional outcome was excellent in both groups, including the patients who underwent plasma exchange due to the steroid refractory ON, confirming results of earlier studies [14, 22–24]. High age-related cortical neuroplasticity may account for this exceptional visual recovery in children, as previously reported by us and others [14, 22, 25].

We observed a significantly more profound global peripapillary atrophy in MOGAD^ped^ in comparison to MS^ped^. The average pRNFL thickness in both groups corresponded well with previous studies of MS-ON with 80.8–95.0 µm and MOGAD-ON with 63.1–76.8 µm. [13, 14, 26–32]. Substantial neuroaxonal retinal atrophy in MOGAD has been reported previously; however, due to an actively relapsing course of MOGAD-ON, most of the patients were investigated after several ON episodes [13, 28, 33]. To investigate a potential predominant atrophy pattern, we focused this study on patients after the first ON attack. Similar to adults a predominantly temporal atrophy could be seen in MS^ped^, while MOGAD^ped^ showed a moderate to profound pRNFL thinning in all peripapillary segments already after the first ON [13, 34–36]. Differences in the pRNFL thinning were most obvious in the nasal peripapillary segments, remaining within normal range in almost all MS-ON eyes. Macular OCT revealed no significant differences in any layer but for mRNFL. This contrasts with several earlier studies showing significant differences in GCIPL thickness, probably due to accumulation of neuroaxonal degeneration after several ON in relapsing disease. VEP latency delay was more pronounced in our MS cohort in comparison to a previous study conducted in pediatric MS (124.6 ± 15.2 for our cohort vs. 115.0 ± 20.0 ms in a previous study) probably due to a selection bias and recruitment of patients with more aggressive disease course, mostly requiring highly effective immunotherapies [37]. Nevertheless, we found a significant P100 latency delay (> 2SD) in only 14% and 29% of MOGAD and MS-ON eyes, demonstrating its relative low diagnostic sensitivity in children during a stable disease stage (> 6 months after first acute ON). In contrast, OCT was substantially more informative in our cohort, among all parameters, significant global pRNFL decrease in MOGAD-ON (90%) and N/T ratio increase in MS-ON (66%) revealed to be most sensitive for the history of ON which corresponds well with a similar study comparing adult NMOSD and MS patients [38].

High diagnostic accuracy of the nasal pRNFL (followed by macular RNFL and global pRNFL) in distinguishing between MOGAD and MS is the most important and clinically relevant finding of our study. OCT can be applied as a rather simple and feasible additional diagnostic tool, especially in patients with a borderline result of antibody testing or those with an unclear disease history, as OCT changes are stable after the initial attack without fluctuations. In contrast, the MOG antibody titer may decrease in the course of the disease.

In our study, we specifically focused on the OCT changes. A composite score, including additional parameters characteristic for MOGAD (e.g., simultaneous bilateral ON, negative oligoclonal bands in cerebrospinal fluid) could be another even more promising approach. Further studies with a larger number of patients are needed to evaluate the diagnostic accuracy of such a score precisely.

We suppose that the observed differences in the atrophy patterns reflect different etiopathogenesis in both diseases. Previous studies have indicated that the temporal predominance in MS may be explained by mitochondrial dysfunction, especially critical for smaller and thinly myelinated parvocellular axons of the PMB that are more vulnerable to the oxidative stress [14, 35, 39]. In contrast, these mechanisms seem to be less relevant for MOGAD-ON, while a massive acute primary MOG-IgG related inflammation involves all distal parts of the optic nerve, additionally resulting in a papilledema and often more severe retroorbital pain [34, 40, 41]. Further research on immunopathogenesis and neuropathological data is needed to investigate the MOG-IgG related optic nerve inflammation and a role of mitochondrial dysfunction in MS precisely.

Due to the retrospective approach, the study is prone to several kinds of potential biases, including selection and reporting bias, especially regarding ON history, as well as heterogeneity of treatment schedules and incomplete data. The sample size was limited due to recruitment difficulties in pediatric patients at a predefined timepoint after the first ON. However, the inclusion of patients after several ON episode could confound data on disease specific atrophy pattern in MOGAD. There were significant differences in the age and sex distribution, as expected based on the different natural course of diseases. Previous studies revealed no age- or sex-dependent difference in the pRNFL thickness in children from 5 to 15 years old [42]. The time between ON and examination was also slightly different between pediatric MOGAD and MS patients. However, considering the observed substantial differences in OCT findings between groups, we suggest that possible minor variations should not be critical for the study results and can be neglected. A real prevalence of the steroid refractory ON in pediatric MS and MOGAD is unknown, but we suppose that it could be increased in our cohort due to patients’ recruitment in specialized neuroimmunological departments. To evaluate a possible effect of this selection bias, we performed a subgroup analysis, by excluding plasma exchange patients. We could confirm the best diagnostic accuracy of three already identified parameters (pRNFL N, pRNFL G, mRNFL), moreover, its sensitivity and specificity turned out to be even higher in this subgroup of patients. VEP data were not available for all patients and sample size was limited. Further larger studies with comparable age and sex distribution are needed to confirm our findings. Moreover, studies in adult MOGAD and MS patients are needed to clarify if the observed disease-dependent retinal atrophy pattern and the applicability of nasal pRNFL as the parameter with the highest accuracy in distinguishing between MOGAD and MS is universal or age-specific.

## Conclusion

After a single ON pediatric MOGAD, patients develop severe global atrophy of the peripapillary RNFL compared to predominantly moderate temporal pRNFL thinning in pediatric MS. Nasal pRNFL enables a diagnostic differentiation between MOGAD-ON and MS-ON in children with a high accuracy and may be used as a supportive diagnostic tool. Moreover, OCT seems to be more sensitive compared to VEP in identification of eyes with a history of ON. Further studies are needed to confirm applicability of OCT in in distinguishing between MOGAD and MS.

## Data Availability

The data that support the findings of this study are available from the corresponding author upon reasonable request.
